# Paracelsus and Hahnemann

**Published:** 1895-11

**Authors:** B. Fincke

**Affiliations:** Brooklyn, N. Y.


### PARACELSUS AND HAHNEMANN.

B. Fincke, M. D., Brooklyn, N. Y.*

In the works of Paracelsus (Genevæ De Tournes 1658 folio, Tractatus II de Astronomia, the following remarkable statement is found : "*In bursa pastoria virtus inest sistendi sanguinem dysenteriae et menstrui. Jam vero eidem virtus quoque inest, fluxum ventris concitandi, et sanguinem non consistendi, sed sapius etiam promovendi. Et sic ves aliae quoque sunt, quae purgant, et saepe restringunt aut quae restringunt et tamen purgant, adeoque hoc modo adversa et contraria apparent. Hujus quae causa? Solius coeli, quod in homine taliter existit, in hoc tale, in alio aliud, quod hunc ita inclinat alium, aliter, adesque in diversis diversimode medicinam perficit. Nam in deductione coeli, prout eas concordare facit ac conjungit, operationes ac virtutes Medicinae universae positae sunt. Si probe non concedant, propositum tuum non succeded.*" This, rendered in English, reads as follows: "In Bursa-pastoria resides the virtue of staying the blood in dysentery and menstruation. However, there also resides in it the virtue of exciting the abdominal flux and of not staying the blood but often also promoting it. And thus there are also other things which purge and often constipate, or which constipate, and yet purge, therefore in this manner appear to be adverse and contrary. What is the reason of this? Only the heaven which in one man exists in this wise, in another in that wise, in another otherwise, and therefore in different individuals perfects the medicine in a different manner. For by deduction from the heaven, according as it makes them concordant and combines them, the operations and virtues of the universal Medicine are determined. If they do not well agree, thy intention will not succeed."

---

* Read before the International Hahnemannian Association, 1895.

This curious passage from Paracelsus exonerates Hahnemann at once from the imputation of having reproduced his Homœopathy from the writings of this wonderful doctor and savant of the Middle Ages. It has become the fashion this late day to cast this blot of plagiarism upon his fair name, even by some who claim to be homœopaths and Hahnemannians, which is unjust and ought to be refuted.

Paracelsus wonders at the fact that Bursa-pastoris stops and promoted bleeding, and that other medicines also present this contrariety of action in other affections, and naturally asks the reason why. But he can give no other reason than that the heaven (*coelum*) exists in the organisms receiving the medicine in this wise or otherwise, and adds the warning, if the ordination of heaven in the sick do not conjoin and combine them—by which the virtues of the universal Medicine are determined —*i. e.*, if they do not agree, *thy* intention of healing will come to naught. If there is any sense in that conclusion which seems to be as obscure as so many other things in the Middle Ages, it is the assumption of a universal Medicine which acts differently in different persons according as the heaven exists in this or the opposite manner. It seems Paracelsus places the action of medicine in the organism whilst the universal Medicine is an obscure power which has the obscure action to react upon it according as the heaven in one or another man exists. There is an obscure presentment in the idea when he uses the word "*concedant*" or " agree," because it points to what Hahnemann two and one-half centuries later brought to light by his discovery of the curativeness of the similars.

Now Paracelsus has, according to the high authority, Rademacher (*Rechtfertigung, etc.*, I, p. 87), rejected the old sentence, *contraria contrariis*—very plainly, because he could not explain why the contrarious action in medicines occurs. Rademacher continues (*l. c.*), " that however he should have put in its place the sentence equals are cured by equals, has indeed been maintained even in our days, but is entirely untrue." It must be remarked that in Paracelsus' time the German " gleich " (English, equal like), was in Latin " *similis* " but not " *equalis*," nor

"*par*," and therefore, as he has not explained the contrarious action of medicines by the "*similia*," he could not be considered the originator of Hahnemann's *Similia Similibus Curantur*.

Paracelsus evidently was not aware of the reason why he rejected the old sentence of Galen *contraria contrariis;* he did it probably on empirical grounds, for in his *Tractatus Taragrani* he wrote: "*Contraria contrariis*—*i. e.*, not drives away cold, etc. This is false, and has never been true in medicine, but it is so: Arcanum and disease, these are the *contraria*. Arcanum is the health, and the disease is adverse to the health; these two are the adverse ones which drive each other away." The real reason, however, depends upon a logical fault which has not been pointed out and cleared up until within a few years. There is a logical and a dynamical contrariety. The confusion of mingling the two concepts is the cause why the action of medicines has been so little understood. It is said: Opium makes sleepy, Opium makes wakeful. Clearly, this is a contrariety, but only a logical one depending upon an induction from a number of facts observed on many different individuals. It makes sleepy in this individual and wakeful in that. Or Bursa-pastoris stops the bleeding in one and promotes it in another. Or it purges in one and constipates in another, etc.

These are all logical contrarieties of action of these medicines. But here is a person whom you want to put to sleep, so you give Opium, which has the property of making sleepy, and if the medicine and the organism agree, as Paracelsus has it, the person will sleep. But if the person after Opium will not sleep, but be wide-awake, even more so than before, your intention, as Paracelsus predicts, will be frustrated, because the heaven existing in that person does not agree with the medicine as it perfects it in a different manner. Paracelsus stands by looking at the curious fact, but cannot explain it. Now here Hahnemann steps in and says: "The person in want of sleep is excited and does not need the medicine which has the contrary action of producing sleep, but a medicine, which under the concurrence of the other symptoms, has the property of keeping wide-awake. For this medicine which is contrary only in a logical sense, is not the medicine which is here needed, viz.: which is contrary to the given case on the dynamical sense. That only is contrary and will heal according to the necessary equality of action and reaction, which is similar to the present state of disease to be turned into its contrary health. In one word, it is only the *simile-contrarium* which will do the business of healing, for if the *contrarium* is no *simile*, it will not be contrary to the state of the organism, and fail to meet the symptoms to be equalized and turned into symptoms of health.

Hence the reason why Paracelsus has been answered by Hahnemann, and it redounds to his eternal glory.

